# d-Chiro-Inositol improves testosterone levels in older hypogonadal men with low-normal testosterone: a pilot study

**DOI:** 10.1186/s12610-021-00146-4

**Published:** 2021-11-12

**Authors:** Maurizio Nordio, Philip Kumanov, Alfonsina Chiefari, Giulia Puliani

**Affiliations:** 1 The Expert Group on Inositol in Basic and Clinical Research (EGOI), Rome, Italy; 2 Department of Experimental Medicine, Sapienza University, Rome, Italy; 3 Medical Faculty, University Hospital of Endocrinology and Gerontology Ivan Penchev, Sofia, Bulgaria; 4 Oncological Endocrinology Unit, IRCCS Regina Elena National Cancer Institute, Rome, Italy

**Keywords:** Male ageing, Functional hypogonadism, Late-onset hypogonadism, Testosterone, Aromatase, D-chiro-inositol, Inositol, Sexual dysfunction, Vieillissement masculin, Hypogonadisme fonctionnel, Hypogonadisme tardif, Testostérone, Aromatase, D-chiro-inositol, Inositol, Dysfonction sexuelle

## Abstract

**Background:**

Several recent journal articles report that d-chiro-inositol (DCI), primarily known as insulin second messenger, influences steroidogenesis. In particular, new evidence is arising on DCI ability to regulate aromatase expression and testosterone biosynthesis. In this regard, DCI administration could represent a good therapeutic opportunity in case of reduced levels of testosterone.

Older men generally have lower testosterone concentrations than younger men, and recent randomized controlled trials have examined whether testosterone treatment might improve health outcomes in this age group. There is limited information about the safety of testosterone replacement therapy in these men, hence DCI could represent an interesting alternative for future trials. Therefore, this study aims to evaluate the effect of DCI treatment on testosterone levels in older male patient.

**Results:**

Ten older men with basal low testosterone levels were enrolled in this study. Patients took 600 mg of DCI, two-times per day, for 30 days. We evaluated hormonal and glycaemic parameters, weight, waist circumference, and Body-Mass Index at baseline (T0) and after 30 days (T1). Finally, all patients also filled in the standardized International Index of Erectile Function questionnaire and performed the Handgrip test at T0 and T1. Men receiving DCI showed increased androgen and reduced oestrogen concentrations, and improved glycaemic profiles. DCI was also associated with reduced weight, Body-Mass Index, waist circumference, and improved grip strength and self-reported sexual function. All these effects led to the improvement of sexual function and physical strength.

**Conclusions:**

In this pilot study, DCI treatment improved the levels of testosterone and androstenedione at the expense of oestrogens in elder men with low basal levels of these hormones without adverse effects.

**Trial registration:**

Clinicaltrials.gov: D-chiroinositol Administration in Hypogonadal Males, NCT04708249

## Résumé

**Contexte:**

Plusieurs articles de revues récents rapportent que le D-chiro-inositol (DCI), principalement connu sous le nom de second messager de l’insuline, influence la stéroïdogenèse. En particulier, de nouvelles preuves apparaissent sur la capacité du DCI à réguler l’expression de l’aromatase et la biosynthèse de la testostérone. À cet égard, l’administration de DCI pourrait représenter une bonne opportunité thérapeutique en cas de réduction des taux de testostérone. Les hommes d’âge avancé ont généralement des concentrations de testostérone plus faibles que celles d’hommes plus jeunes, et de récents essais contrôlés randomisés ont examiné, si le traitement par testostérone pourrait améliorer dans ce groupe d’âge les résultats en matière de santé. Il existe peu d’informations sur l’innocuité de cette thérapie de remplacement de la testostérone chez ces hommes ; le DCI pourrait ainsi constituer une intéressante alternative pour de futurs essais. Par conséquent, la présente étude vise à évaluer l’effet du traitement par DCI sur les taux de testostérone chez les hommes d’âge avancé.

**Résultats:**

Dix hommes d’âge avancé ayant un faible taux basal de testostérone ont été recrutés dans la présente étude. Les patients ont pris 600 mg de DCI, deux fois par jour, pendant 30 jours. Nous avons évalué les paramètres hormonaux et glycémiques, le poids, le tour de taille et l’indice de masse corporelle au départ (T0) et après 30 jours (T1). Enfin, tous les patients ont également rempli le questionnaire normalisé de l’indice international de la fonction érectile et ont effectué le test de force de préhension (handgrip test) à T0 et T1. Les hommes prenant le DCI ont présenté des concentrations augmentées d’androgènes et réduites d’œstrogènes, ainsi qu’une amélioration des profils glycémiques. La prise de DCI était également associée à une réduction du poids, de l’indice de masse corporelle, du tour de taille, et à une amélioration de la force de préhension et de la fonction sexuelle autodéclarée. Tous ces effets ont conduit à une amélioration de la fonction sexuelle et de la force physique.

**Conclusions:**

Dans cette étude pilote, le traitement par DCI a amélioré les taux de testostérone et d’androstènedione au détriment des œstrogènes chez des hommes d’âge avancé ayant de faibles taux de base de ces hormones, sans effets indésirables.

## Background

Male hypogonadism (MH) is a pathological condition characterized by low testosterone levels and spermatogenesis impairment, due to abnormalities in hypothalamic-pituitary-testicular (HPT) axis. Hypogonadism can be either primary, with abnormalities in HPT axis at testicular level, or secondary, displaying abnormalities in the signal from the hypothalamus or the pituitary gland [1, 2]. Older men have lower testosterone concentrations compared to younger men, but the role of testosterone treatment in the absence of pituitary or testicular disease is uncertain [3–7]. This age-dependent decline in testosterone levels is highly variable and likely related to adiposity, medications and chronic diseases. The main symptoms and signs of androgen deficiency include reduction of sexual desire and spontaneous erections, loss of body hair, subfertility and erectile dysfunction [8–11]. Among hypogonadal older men, some can also display increased levels of luteinizing hormone (LH), a condition called compensated hypogonadism [12].

Nowadays, the testosterone replacement therapy (TRT) is universally accepted as the main treatment for pathological hypogonadism in men, but its role in functional hypogonadism is unclear [1, 7, 13, 14]. However, little is known about the potential adverse events related to this therapy in older men, especially on prostate health and on cardiovascular system [7, 15]. Therefore, considering the importance of restoring physiological testosterone levels in the plasma of these patients and the lack of reliable information about TRT safety, new safe treatments should be found [7]. Targeting aromatase, the enzyme that converts androgens into oestrogen, can represent a new therapeutical opportunity. Aromatase, also called CYP19A1, specifically converts androstenedione and testosterone into their respective oestrogens oestrone and oestradiol [16]. Because both the substrates and the products are important hormones, alterations in the expression or in the activity of the enzyme strongly impact physiology. Besides, aromatase inhibitors are nowadays widely used in patients with low testosterone/oestradiol ratio (T/E) [17].

Interestingly, Sacchi et al. [18] demonstrated that the treatment of granulosa cells with d-chiro-inositol (DCI) reduces the expression of aromatase in a dose-dependent manner. DCI belongs to the family of inositol, natural cyclic polyols essential for cellular trafficking. They are either introduced with the diet or synthesized *de novo* starting from glucose-6-phosphate. In normal physiology, DCI is the second most diffused isomer in mammals, and it is synthesized by an epimerase enzyme from its most represented relative, myo-inositol (MI). Both MI and DCI play crucial role at cellular level, being involved in intracellular mechanisms of signal transduction [19, 20]. Indeed, they are second messengers of insulin, even if they play different roles in this process. MI improves cellular glucose absorption, while DCI stimulates glycogen synthase and activates pyruvate dehydrogenase (PDH), thus supporting ATP production by the oxidative metabolism of glucose via Krebs cycle. Overall, the correct cellular ratio between MI and DCI is fundamental to optimize glucose uptake and its metabolism [20–22]. Summarizing, both DCI [23–26] and MI [25–28] are insulin sensitizer compounds, even if they exert different roles.

Before Sacchi et al., other authors already reported the involvement of DCI in steroidogenesis. Indeed, Nestler et al. demonstrated that insulin induces testosterone biosynthesis in cultured thecal cells from women with Poly Cystic Ovaries Syndrome (PCOS) via DCI-containing inositol-phospho-glycans (IPGs) [29]. On the contrary, insulin is known to stimulate testosterone production in human Leydig cells [30, 31]. As a consequence, treatment with DCI may likely mimic the effects of insulin stimulus. Recently, Monastra et al. investigated the properties of DCI in raising testosterone in healthy men, reporting a 24.3 % increase in testosterone levels [32].

On these premises, DCI could represent a safe and interesting alternative to TRT [33]. Hence, we evaluated the efficacy of this natural molecule to restore physiological testosterone concentrations in older men with low-normal levels, with special attention to possible adverse effects.

## Patients and methods

### Study design and intervention

We registered this study on Clinicaltrials.gov (ID NCT04708249). In this pilot study, we enrolled 10 elder men with decreased sexual desire, weakened morning erections, erectile dysfunction and a morning level of serum testosterone in the range 8–11 nmol/L [34] from October 2019 to January 2020. All subjects involved provided written Informed Consent before participation and the study was carried out according to the Declaration of Helsinki. We considered as inclusion criteria: age range 65–75, insulin resistance defined as Homeostatic Model Assessment for Insulin Resistance Index (HOMA-IR Index) > 2.5, and BMI between 25 and 30. We considered as exclusion criteria: alcohol intake and/or drug abuse, recent hormonal treatment, smoking, obesity, systemic or endocrine diseases, male accessory gland infection, a clinical history of cryptorchidism or varicocele and micro-orchidism. We treated the patients with 600 mg of DCI, two times per day on an empty stomach, for 30 days. We evaluated for all patient weight, BMI, insulin, glycaemia, HOMA Index, serum testosterone, androstenedione, LH, prolactin, oestradiol (E2), oestrone, and T/E at baseline (T0) and after 30 days (T1). Waist circumference was measured by the same independent operator external to the study. Moreover, all patients were required to fill in the standardized International Index of Erectile Function (IIEF) questionnaire to evaluate erectile dysfunction [35] at T0 and T1. Finally, to evaluate treatment efficacy on muscle strength, patients performed the Handgrip test at T0 and T1. Handgrip dynamometers were calibrated before every test, and all examiners were well trained in the test procedures [36]. Patients used the dominant hand to squeeze the handle as hard as possible for 3–5 s. Using a hand-held dynamometer (Good Strength, IGS01, Metitur Oy, Jyväskylä, Finland), patients were seated with elbow flexed at 110°. We repeated measurements on the same subject after a recovery period of 30 s. If two results differed by more than 10 %, a third trial was carried out [37].

### Sample collection and preparation

Blood samples were collected by venepuncture at baseline and after 30 days of DCI treatment. Blood samples were centrifuged, and serum was stored at − 20 °C until assayed.

### Serum assays

Serum levels of insulin, glycaemia, testosterone, androstenedione, oestrone, oestradiol, and LH were measured via ELISA using commercial kits. Serum prolactin measurement were performed via RIA. The following standard ranges were used as reference for the analyses: testosterone 270–1000 ng/dL; oestradiol 20–45 pg/mL; oestrone 10–80 pg/mL; glycemia 80–115 mg/dL; insulinemia 1.9–25.0 µIU/mL; LH 1.8–14.6 IU/L; prolactin 1–20 ng/mL.

### Statistical analyses

Statistical analyses through Shapiro-Wilk test confirmed the normal distribution of the values. We therefore indicated mean ± standard deviation as representations of results. The statistical values derive from Student’s two-tailed paired T-test. A *p*-value < 0.05 was considered significant. Considering the elevated number of statistical variables analysed, we performed a correction of the p-values via Holm-Bonferroni method.

## Results

All 10 patients (mean age of 69.3 ± 3.6 years) correctly completed this pilot study. Table 1 shows that DCI significantly rebalanced the hormonal profile, increasing serum testosterone and androstenedione, and reducing oestradiol and oestrone. As consequence, the T/E ratio increased with great significance. In addition, we observed a significant decrease in levels of LH after the treatment. We also found significant improvements in glycaemic profile following DCI intervention. In particular, the treatment reduced glycaemia and insulin resistance, as indicated by the significant reduction of the HOMA-IR Index. The administration of DCI also induced statistically significant reduction of waist circumference, BMI and weight. The patients reported a statistically significant enhancement (*p* < 0.05) in sexual function and physical strength. Throughout the study, we did not observe adverse events, confirming the high safety profile of DCI.

**Table 1:** Parameters analysed in ten patients at baseline and after 1-month treatment

Patients characteristics		Baseline values	T1 values	*p*-values	*Holm-Bonferroni method*
Age (years)		69.30 ± 3.62			
Height (cm)		175.80 ± 5.51			
Weight (Kg)		86.04 ± 6.91	84.69 ± 6.56	0.002	0.004
BMI (kg/m^2^)		27.81 ± 1.32	27.38 ± 1.33	0.001	0.003
Waist Circumference (cm)		115.80 ± 4.64	110.80 ± 4.85	0.003	0.004
HOMA-IR Index		5.41 ± 1.71	3.52 ± 1.02	0.001	0.003
Glycaemia (mg/dL)		103.10 ± 11.14	91.10 ± 6.89	0.001	0.004
Insulin (µIU/L)		21.34 ± 6.27	15.64 ± 4.29	0.004	0.006
Testosterone (ng/dL)		222.80 ± 5.59	262.80 ± 32.11	0.009	0.007
Androstenedione (ng/mL)		0.43 ± 0.12	0.88 ± 0.23	0.002	0.004
Oestradiol (pg/mL)		42.24 ± 4.32	34.15 ± 5.36	0.018	0.007
Oestrone (pg/mL)		128.59 ± 5.60	93.59 ± 13.67	0.001	0.003
T/E2 Ratio		5.31 ± 1.11	7.83 ± 2.04	0.019	0.031
LH (mIU/mL)		12.61 ± 2.67	9.76 ± 1.64	0.005	0.007
Strength Test (Kg)		24.98 ± 1.74	27.10 ± 2.07	0.004	0.005
IIEF Questionnaire		11.50 ± 1.96	13.60 ± 1.96	0.001	0.003

Characteristics of patients involved in the study at baseline and after one-month treatment. Data are shown as mean ± SD. The statistical analysis are performed via Student’s paired T-Test and fixed via Holm-Bonferroni method

*T1* after 30 days of treatment; *BMI* Body-mass Index, *HOMA-IR* Homeostatic Model Assessment for Insulin Resistance, *T/E2* Testosterone/Oestradiol ratio, *LH* Luteinizing Hormone, *IIEF* International Index of Erectile Function

## Discussion

Despite the number of studies on DCI, there is lack of information about its clinical effects on steroidogenesis in men. However, *ex-vivo* evidence exists regarding the effect of DCI on aromatase and on testosterone biosynthesis, but they need clinical validation. In this open-label uncontrolled study, we report that the intake of 1200 mg/day DCI for 30 days restores physiological hormone levels in elder hypogonadal men and improves muscle strength and self-reported erectile function. The reader should take into account that the data on erectile function derive from self-reported values and thus could be biased.

Table 1 shows that the treatment with 1200 mg/day DCI for 30 days in elder men with low testosterone and androstenedione significantly raised the levels of these hormones at the expense of oestradiol and oestrone. Remarkably, DCI also improved LH, inducing a reduction toward physiological levels, as previously described in women [38]. Men receiving DCI showed an increase in testosterone and androstenedione concentrations, and a reduction in oestrogen and LH concentrations. We speculate that this could reflect a testicular effect with appropriate feedback regulation at the pituitary level.

Our data also highlight the effect of DCI on insulin signalling, with reduction in HOMA-IR Index, plasma insulin and glycaemia, indicating an improvement of glycaemic profile. Interestingly, patients reported an improvement in waist circumference, weight and BMI. A possible explanation could be the recovery of normal glycaemic and hormonal profiles, but also to the increased muscle strength, as depicted in Table 1. Such concomitant improvement in waist circumference and in muscle strength suggest a reduction in fat mass coupled with a similar increase in muscle mass. As expected, all these results are reflected by the improvement in self-reported erectile function, confirming the clinical efficacy of DCI treatment in hypogonadal elder men.

The clinical features of the patients enrolled in this study match the diagnostic criteria of Late-Onset Hypogonadism (LOH). This condition is a pre-pathological state of some elder males, characterized by a decline in serum testosterone levels, accompanied by several symptoms including: reduction in sexual activity, subfertility, erectile dysfunction, reduced physical performance, decreased energy and motivation, depressed mood, sleep disturbances, reduced muscle mass and strength and increased body fat. Although several authors recognize the importance of LOH, the actual categorization of LOH as a well-defined pathology is still controversial [12]. Nevertheless, hypogonadism is a well-defined and widely recognized issue concerning male fertility. Indeed, at least two different typologies of hypogonadism exist, even if the diagnosis of a patient may be controversial. Hypogonadism is divided into primary and secondary, however, hybrid conditions exist, and they go under the name of mixed hypogonadisms [39]. These conditions are generally considered to be part of secondary hypogonadism, due to the hormonal/metabolic causes and the lack of testicular damage. Mixed hypogonadisms include a clinical picture generally referred to as compensatory hypogonadism, a particular subtype of LOH, featuring advanced age, low testosterone and high LH in the absence of testicular damage [12]. The population analysed in this study match the criteria of the compensatory hypogonadism. Nevertheless, such theorical subdivisions are not always recognizable in clinical practice, and therefore their clinical utility is unclear.

Although TRT is universally considered the main treatment in all the subtypes of elder hypogonadal men, the lack of information about safety opened the debate about the cost/benefit of this therapy in elder men [15]. Indeed, increased cardiovascular and prostate risk should be considered in older men under hormonal replacement treatments. On the contrary, DCI is a safe molecule that impacts steroidogenesis, restoring physiological levels of hormones in case of reduced androgens or increased oestrogens. To the best of our knowledge, this is the first report of DCI effect in raising androgen levels in men with impaired signalling along the HPT axis.

The properties of DCI to alter the steroidogenesis were firstly described by Nestler in the early 90’s [40]. Since then, only few works have focused on the impact of DCI on steroidogenesis [18, 29]. In fact, most of the information available on DCI concerns its properties as insulin sensitizer compound. Despite the first evidence of its effect on aromatase, DCI is still used to improve the clinical conditions of PCOS patients, regardless of their androgen status and posology [22]. Besides, in men, DCI is likely to play pivotal role at testicular level. In fact, DCI is a second messenger of insulin, which stimulates testosterone biosynthesis in Leydig cells [30, 31]. Our findings seem to indicate that such activity of DCI on androgen levels may be stronger than originally supposed. As a consequence, physicians should consider this effect when recommending DCI supplementation.

Further studies demonstrated that the treatment with insulin and insulin-like growth factor I and II reduced the expression of aromatase. Their investigation also highlighted that this modulatory signal is mediated by IPG [40]. Moreover, the results from Sacchi et al. show that DCI has a inhibitory effect on aromatase expression [18]. The finding that the inhibition of aromatase expression is not strictly dependent on IPG highlights that IPGs are not the only inositol-based second messengers of insulin, but also inositol-phosphates play a pivotal role in the transduction of insulin signal [22].

Being this a short-term open-label pilot study, we believe that DCI should be further investigated as a safe alternative to TRT, especially in elder patients who can mostly experience side effects. The principal limitations of the present study were the lack of a control group and thus the impossibility to introduce a placebo as control and to randomize patients. Even if this a short-term study, we want to report that no side effects occurred during this trial, confirming the safety of DCI [33]. For all these reasons, analysing our results, we should point out the positive action of the treatment with 1200 mg of DCI in elder hypogonadal males with low testosterone.

## Conclusions

In this open-label pilot study, older men with low-normal testosterone levels receiving DCI showed improved glycaemic and hormonal profiles, without apparent adverse effects over the 30-day study duration. If our results will be confirmed in stronger randomised and placebo-controlled trials, DCI could be considered as a suitable agent to increase testosterone levels and improve health outcomes in older men with physiological hypogonadism.

## Data Availability

The datasets generated and analysed during the current study are not publicly available due to privacy but are available from the corresponding author on reasonable request.

## Abbreviations

ATP: Adenosine Triphosphate
BMI: Body-Mass Index
DCI: d-chiro-inositol
E2: Oestradiol
HPT: Hypothalamic-pituitary-testicular
IIEF: International Index of Erectile Function
IPGs: Inositol-phospho-glycans
LH: Luteinizing hormone
LOH: Late-Onset Hypogonadism
MH: Male hypogonadism.
MI: Myo-inositol
PCOS: Poly Cystic Ovaries Syndrome
PDH: Pyruvate dehydrogenase
T/E: Testosterone/oestrogen ratio
TRT: Testosterone replacement therapy
